# In Vitro System for Studying Ilhéus Virus, a Neglected Arbovirus: Ultrastructural Characterization of Cytopathology, Morphology, and Morphogenesis

**DOI:** 10.3390/v17030320

**Published:** 2025-02-26

**Authors:** Maycon Douglas do Nascimento Garcia, Igor Pinto Silva Da Costa, Marcos Alexandre Nunes da Silva, Vivian Neuza dos Santos Ferreira, Ana Luisa Teixeira de Almeida, Gabriela Cardoso Caldas, Andressa Santos de Almeida, Ana Maria Bispo de Filippis, Natalia Fintelman-Rodrigues, Aline de Paula Dias da Silva, Marcelo Alves Ferreira, Thiago Moreno L. Souza, Alex Pauvolid-Corrêa, Debora Ferreira Barreto-Vieira

**Affiliations:** 1 Laboratório de Morfologia e Morfogênese Viral, Instituto Oswaldo Cruz, Fundação Oswaldo Cruz, Rio de Janeiro 21040-900, RJ, Brazil; igorcosta@aluno.fiocruz.br (I.P.S.D.C.); marcossilva@aluno.fiocruz.br (M.A.N.d.S.); vivian.ferreira@ioc.fiocruz.br (V.N.d.S.F.); anaalmeida@aluno.fiocruz.br (A.L.T.d.A.); gabrielacardosocaldas@gmail.com (G.C.C.); andressaalmeida143@gmail.com (A.S.d.A.); 2 Laboratório de Biologia Estrutural de Vírus, Instituto de Bioquímica Médica, Universidade Federal do Rio de Janeiro, Rio de Janeiro 21941-902, RJ, Brazil; 3 Laboratório de Arbovírus e Vírus Hemorrágicos, Instituto Oswaldo Cruz, Fundação Oswaldo Cruz, Rio de Janeiro 21040-900, RJ, Brazil; ana.bispo@ioc.fiocruz.br; 4 Laboratório de Imunofarmacologia, Instituto Oswaldo Cruz, Fundação Oswaldo Cruz-Fiocruz, Rio de Janeiro 21040-900, RJ, Brazil; nataliafintelman@gmail.com (N.F.-R.); aline.paula@hotmail.com.br (A.d.P.D.d.S.); thiago.moreno@fiocruz.br (T.M.L.S.); 5 Centro de Desenvolvimento Tecnológico em Saúde, National Institute for Science and Technology on Innovation on Diseases of Neglected Populations, Fundação Oswaldo Cruz, Rio de Janeiro 21040-900, RJ, Brazil; malvesf68@gmail.com; 6 Laboratório de Virologia Veterinária de Viçosa, Departamento de Veterinária, Universidade Federal de Viçosa, Viçosa 36570-900, MG, Brazil; pauvolid-correa@ufv.br

**Keywords:** Ilhéus virus, neglected arbovirus, cytopathology, ultrastructural studies, morphology, morphogenesis

## Abstract

Ilhéus Virus (ILHV) was first detected in 1944 in Ilhéus, state of Bahia, northeast Brazil. During cellular infection, orthoflaviviruses induce cellular changes related both to the replication process, the formation of replication complexes, and to structures resulting from cellular damage. Although more detailed data are available in the literature for other orthoflaviviruses, the relationship between ILHV, the formation of these structures, its replication cycle, and cellular changes remains unknown. One of the main objectives of this study is to characterize the primary ultrastructural changes in green monkey kidney epithelial cell lineage (Vero cell) infected with ILHV, as well as to map its replication cycle, virion structure, and genome. To achieve these objectives, Vero cell monolayers were infected with an MOI of 0.01 and collected at different times post-infection. Cell monolayers were evaluated under bright-field microscopy and transmission electron microscopy. Ultrastructural analyses confirmed that ILHV can induce the formation of double-membrane vesicles, convoluted membranes, and vesicular packets. These structures, like those observed in zika (ZIKV) and dengue (DENV) viruses, form replication complexes that aid ILHV’s replication process in cells. Our preliminary results reveal that ILHV infection induces cytopathogenesis like that observed in vitro studies for other arboviruses.

## 1. Introduction

The Ilhéus virus (ILHV) is an arbovirus that was detected for the first time in the city of Ilhéus, state of Bahia, northeast Brazil [1]. The ILHV is maintained through enzootic cycles of transmission primarily involving birds and mosquitoes. ILHV has been isolated from mosquitoes of different species and biomes of Brazil, such as the Pantanal and the Amazon [2,3]. In serological studies, specific antibodies have been detected in a wide range of vertebrates, including rodents, buffaloes, equids, monkeys, and birds [2,4,5,6,7,8,9]. Cases reported have been reported in Brazil, which concentrates most cases [10,11,12], Bolivia [13], Ecuador [14], Argentina, and Trinidad and Tobago [15]. More recently, an epidemiological study conducted in the Amazon region with 300 military personnel found that more than half had been exposed to ILHV [16].

Clinical infection of ILHV is similar to other arboviral diseases, which makes clinical diagnosis difficult. The lack of rapid tests makes the diagnosis of ILHV infection even more challenging. The cross-reactivity between ILHV and other orthoflaviviruses such as zika and dengue viruses is a limiting factor for the diagnosis in enzootic regions. Despite most ILHV infections being inapparent, when clinical infections cause headache, fever, muscle pain, uveitis, and in rarer cases, the patient’s condition can progress to encephalitis and other neurological symptoms [12,17].

ILHV is currently classified to the genus *Orthoflavivirus* and family Flaviviridae [18]. Its genome is composed of single-stranded positive-sense RNA, and its morphology is spherical with an approximate diameter of 40 nm [19]. The viral genome is translated into a single polyprotein, which is cleaved by both viral and cellular proteases. This cleavage results in the formation of three structural proteins: capsid (C), precursor membrane protein (prM), and envelope protein (E), as well as seven non-structural proteins (NSPs), NS1, NS2A, NS2B, NS3, NS4A, NS4B, and NS5 [19], as observed for other orthoflaviviruses. The rough endoplasmic reticulum (RER) is a crucial organelle in the replication process of orthoflaviviruses and the assembly of virions. During the replication of orthoflaviviruses such as zika and dengue viruses, replication complexes are formed in the RER, functioning with the assistance of non-structural proteins to synthesize viral RNA and assemble viral particles [20].

Research on zika and dengue virus infections in cell cultures has demonstrated cytoplasmic rearrangement in infected cells, mainly involving the remodeling of the RER [21,22,23]. Moreover, these viruses also alter the structures of other organelles, such as the mitochondria and the cell nucleus, to facilitate the viral replication process [22,24,25,26]. Previous studies has demonstrated the susceptibility and permissiveness of mammalian and mosquito cell lines to ILHV infection [19]. Furthermore, ILHV infection in *Aedes albopictus* (C6/36), human, and hamster cell lines results in the production of infectious viral particles with high titers [27]. Although the role of ultrastructural changes is better understood for other orthoflaviviruses, the exact role of these structures in the replication cycle and virion assembly of ILHV remains unclear. In this study, we evaluated the susceptibility and permissiveness of green monkey kidney epithelial cell lineage (Vero cells) for infection by ILHV. Monolayers were experimentally infected with an ILHV and morphologically analyzed by bright-field microscopy and transmission electron microscopy (TEM). ILHV used in this study was isolated from mosquitos *Culex* sp. The successful infection and subsequent morphological alterations observed during the experimental infections corroborates the potential of Vero cells as a cell model for studying ILHV–host interactions. The findings presented here will contribute to a better understanding of the cellular mechanisms involved in ILHV infection, which can ultimately inform guidelines of research and therapeutic strategies.

## 2. Materials and Methods

### 2.1. ILHV Strain

The ILHV strain (GenBank: MK332106.1/SPAR158517) was originally isolated from mosquitos *Culex* sp. in the state of São Paulo, Brazil, in 1994. The sample used in this work was kindly provided by the Arbovirus and Hemorrhagic Virus Laboratory of the Oswaldo Cruz Institute (IOC), Oswaldo Cruz Foundation (Fiocruz), Rio de Janeiro, Brazil. Virus strain was provided for research purpose, in accordance with Resolution 2.998.362 IOC/Fiocruz.

### 2.2. Cell Lineage

For viral propagation and titration, Vero CCL81 (Banco de células do Rio de Janeiro #0245) was used. Cells were supplemented with DMEM high glucose medium (Dulbecco’s Modified Eagle Medium/GIBCO), 10% fetal bovine serum (FSB/GIBCO), and penicillin/streptomycin, 100 U/mL. The monolayers were maintained at 37 °C in a 5% CO_2_ atmosphere. During experimental infections, cells were kept at 3% FBS.

### 2.3. Clarification and Viral Titer

For clarification, the cell monolayer was infected with a multiplicity of infection (MOI) of 0.01, and the supernatant was collected 72 h post-infection (h p.i.). The supernatant samples were centrifuged at 4500 RPM, 4 °C for 30 min. The titration of the supernatants was performed using the Plaque-Forming Unit (PFU)/mL technique. The cells were incubated with virus dilutions for 1 h, after which DMEM with 2% FBS and 2.5% carboxymethylcellulose was added. The plaques were revealed after 96 h, then fixed and stained with crystal violet solution (1% crystal violet, 30% ethanol, 20% formalin, and 49% water). All experimental procedures were performed in a biosafety level 3 (BSL-3) laboratory.

### 2.4. Statistical Analysis

For statistical analysis of titration kinetics, we used the Kruskal–Wallis test and R Core Team (2024), R: A Language and Environment for Statistical Computing, R Foundation for Statistical Computing, Vienna, Austria. <https://www.R-project.org/>, accessed on 15 November 2024.

### 2.5. Infection Kinetics

Aiming at morphological analysis by bright-field microscopy and transmission electron microscopy (TEM), infection kinetics (MOI of 0.01) was performed on the Vero CCL81 cell line (1, 2, 3, 24, 48, 72, and 96 h p.i.). Bright-field microscopy analyses were performed at time intervals between 24 and 96 h p.i and compared with the control condition (uninfected monolayer and with the same culture time). For ultrastructural analyses for TEM, monolayers of all points were collected and processed. Supernatants were collected from all points and titrated using the PFU/mL technique. All experimental procedures were performed in a BSL-3 laboratory.

### 2.6. Analysis of Cytopathic Effects by Bright-Field Microscopy

To evaluate cytopathic effects (CPEs), monolayers of infected Vero lineage cells were observed under an EVOS XL CORE bright field microscope at 24, 48, 72, and 96 h p.i. No type of coloring was applied to the samples.

### 2.7. Processing of Cell Monolayers for TEM Analysis

For analysis of cells infected by the ILHV virus at the ultrastructural level, the monolayers were released from the bottles under the action of trypsin. The resulting cell suspensions were fixed and post-fixed by immersion in a 1% glutaraldehyde solution in sodium cacodylate buffer (0.1 M, pH 7.2) and 1% buffered osmium tetroxide, respectively. Following fixation and post-fixation, the samples were dehydrated through immersion in increasing concentrations of acetone (15%, 30%, and 50% for 15 min each; 70% in 1% uranyl acetate for 30 min; 90% for 5 min; and twice in 100% for 10 min). Subsequently, the cells were embedded in epoxy resin and polymerized at 60 °C for three days [28,29]. Ultrathin sections (50–70 nm) were then obtained from the resin blocks using an ultramicrotome equipped with diamond knives. The sections were collected on uncoated copper grids with a mesh size of 300, positively contrasted in 2% uranyl acetate in 50% ethanol, and lead citrate [30], and examined using a Hitachi HT 7800 transmission electron microscope (Hitachi, Tokyo, Japan).

### 2.8. Quantitative Analysis of Viral Particle Size

Micrographs from distinct fields were acquired with a Hitachi HT 7800 transmission electron microscope (Hitachi, Tokyo, Japan). Subsequently, the diameter measurement of 200 viral particles located in the rough endoplasmic reticulum (RER) lumen was manually performed with the aid of ImageJ software version 1.53t (NIH ImageJ, National Institutes of Health, Bethesda, MD, USA). All data were compiled, and both graph and descriptive statistics were generated in GraphPad Prism software version 8.0.1 (GraphPad Software Inc., La Jolla, San Diego, CA, USA).

### 2.9. Sequencing and Representative Phylogenetic Tree of ILHV

For an unbiased RNA-seq and further metatranscriptomic analysis, total RNA samples were processed using the MGIEasy RNA Library Prep Set (MGI Tech Co., Ltd., Shenzhen, China). In Brief, RNA was first fragmented to a size of 250 bp, which was subsequently converted into double-stranded DNA. The library preparation process included end repair, adaptor ligation, PCR amplification to enhance the overall library yield, denaturation, and circularization of single-stranded libraries. The pooled libraries were then converted into DNA nanoballs using rolling circle amplification and sequenced as paired-end reads (150 nt) on the MGISEQ-G400 platform (MGI Tech Co., Ltd., Shenzhen, China). FASTQ file processing and virome composition were determined using Genome Detective [31], and de novo assembled contigs were compared with reference virus databases (NCBI RefSeq) to obtain similarity indices and assign the species ID.

The ILHV sequence was compared with representative genomes deposited in GenBank, and a phylogenetic tree was constructed in MEGA 11 [32] with a total of 1000 bootstraps. The evolutionary history was inferred by using the Maximum Likelihood method and General Time Reversible model [33]. The tree with the highest log likelihood (−21,584.68) is shown. The percentage of trees in which the associated taxa clustered together is shown next to the branches. A discrete Gamma distribution was used to model evolutionary rate differences among sites (five categories (+G, parameter = 0.1274)). The tree is drawn to scale, with branch lengths measured in the number of substitutions per site. This analysis involved 13 nucleotide sequences. There was a total of 10.760 positions in the final dataset.

## 3. Results

### 3.1. Sequencing of ILHV

**Figure 1:**
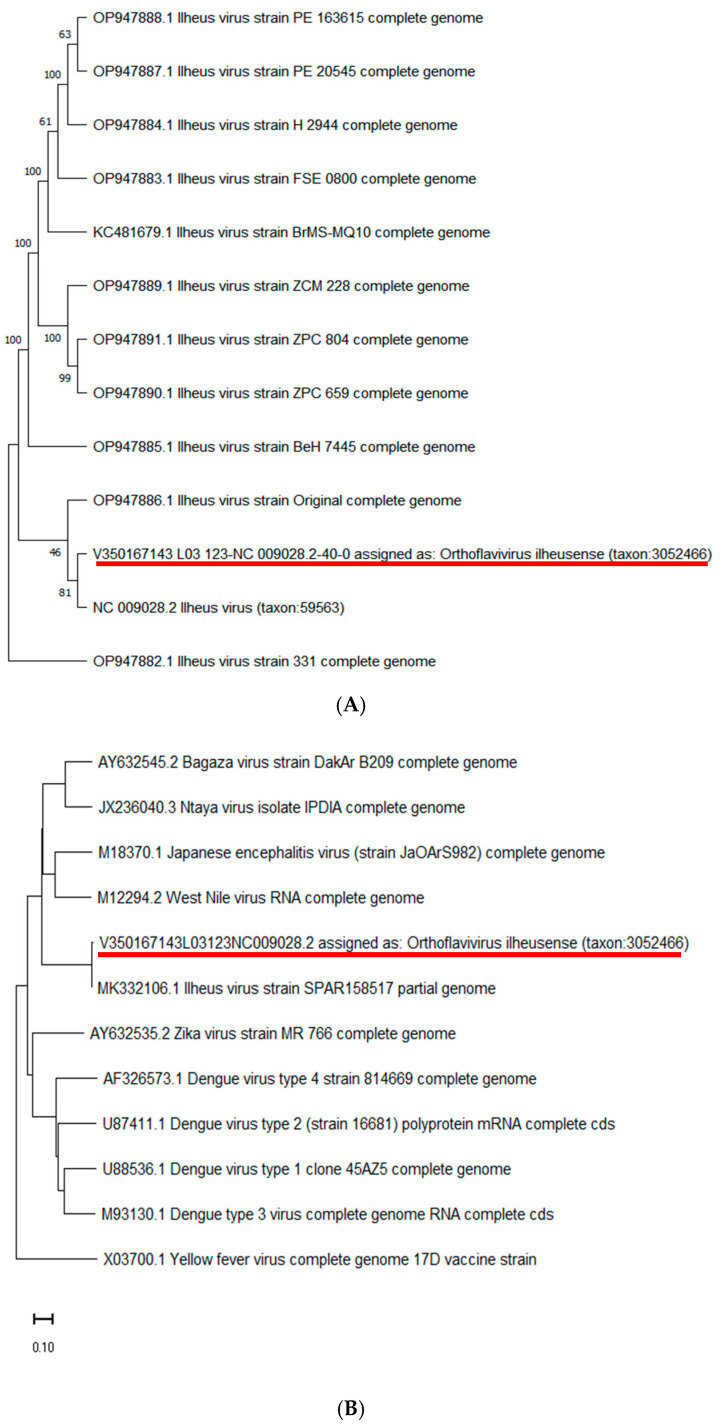
Phylogenetic analysis of ILHV. Viral RNA was sequenced using the NGS method, and the sequence was compared with other ILHV strains (**A**) and with other orthoflaviviruses (**B**). The red line indicates the virus sequenced in this work.

ILHV was passed five times in Vero cells. To evaluate genetic stability, the supernatant from infected cells was sequenced to compare the ILHV sequence with other orthoflaviviruses and different strains of the virus (Figure 1). The sequence of the ILHV studied here (GenBank accession code #PQ846503) is indeed phylogenetically related to the original isolate (Figure 1).

### 3.2. Morphological Alterations of Infected Cells

**Figure 2:**
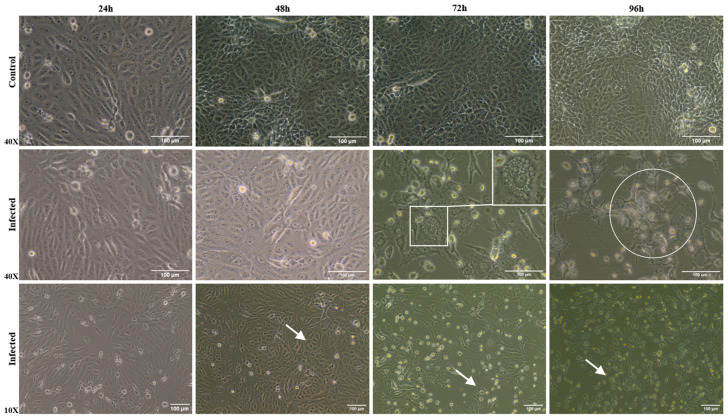
Cytopathic effects in Vero cells infected with ILHV analyzed by bright-field microscopy at different times post-infection. Monolayers were compared with control and ILHV-infected conditions at different kinetic times (24, 48, 72, and 96 h p.i.) are observed by different objective lenses. Note: in infected monolayers, cells with rounded shape (white arrow) and presenting vesicles in the cytoplasm (square) and cell death (circle).

Brightfield microscopy images were taken to evaluate whether the mammalian cell line (Vero CCL81 cells), when infected by ILHV, exhibits CPEs. According to our analyses, up to 48 h p.i., it was not possible to observe CPEs. From 72 h p.i. onwards, CPEs became more evident, with cells exhibiting a more rounded shape and a higher rate of cell death (Figure 2). At 96 h p.i., the cells showed the most pronounced CPEs compared to the control condition. Even in bright field microscopy analyses, it was possible to determine the presence of more detailed structures and vesiculated cells when infected.

### 3.3. Infectious Viral Particles in the Supernatant of Vero Cells

**Figure 3:**
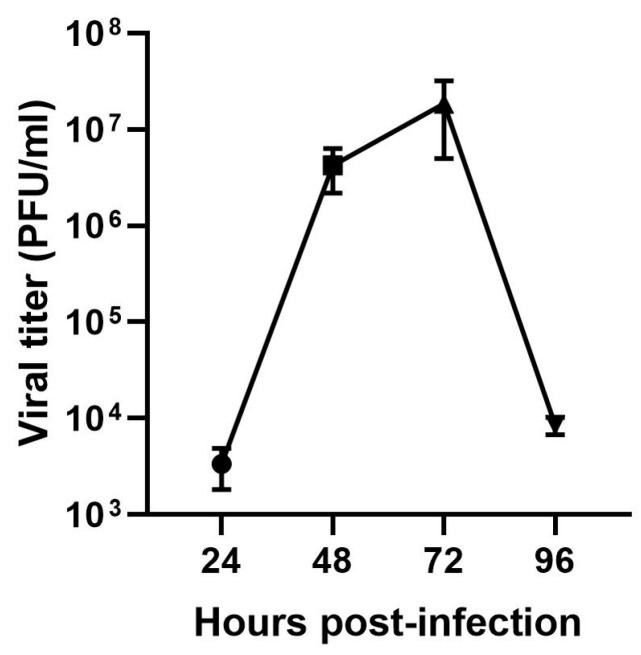
Titration of infection kinetics in Vero cells from 24 to 96 h post-infection. *Y*-axis = viral titer (PFU/mL), *X*-axis = hours post-infection (*n* = 4).

To assess the infectivity of the supernatant from infected cultures, the supernatant from all kinetic points was collected and titrated. Only points from 24 h p.i. showed quantifiable viral titers. The peak infectivity of ILHV in Vero cells occurred on the third day, with an average of 1.85 × 10^7^ PFU/mL and a maximum value of 3 × 10^7^ PFU/mL (Figure 3).

### 3.4. Ultrastructural Analyses by TEM of Infected Cells

**Figure 4:**
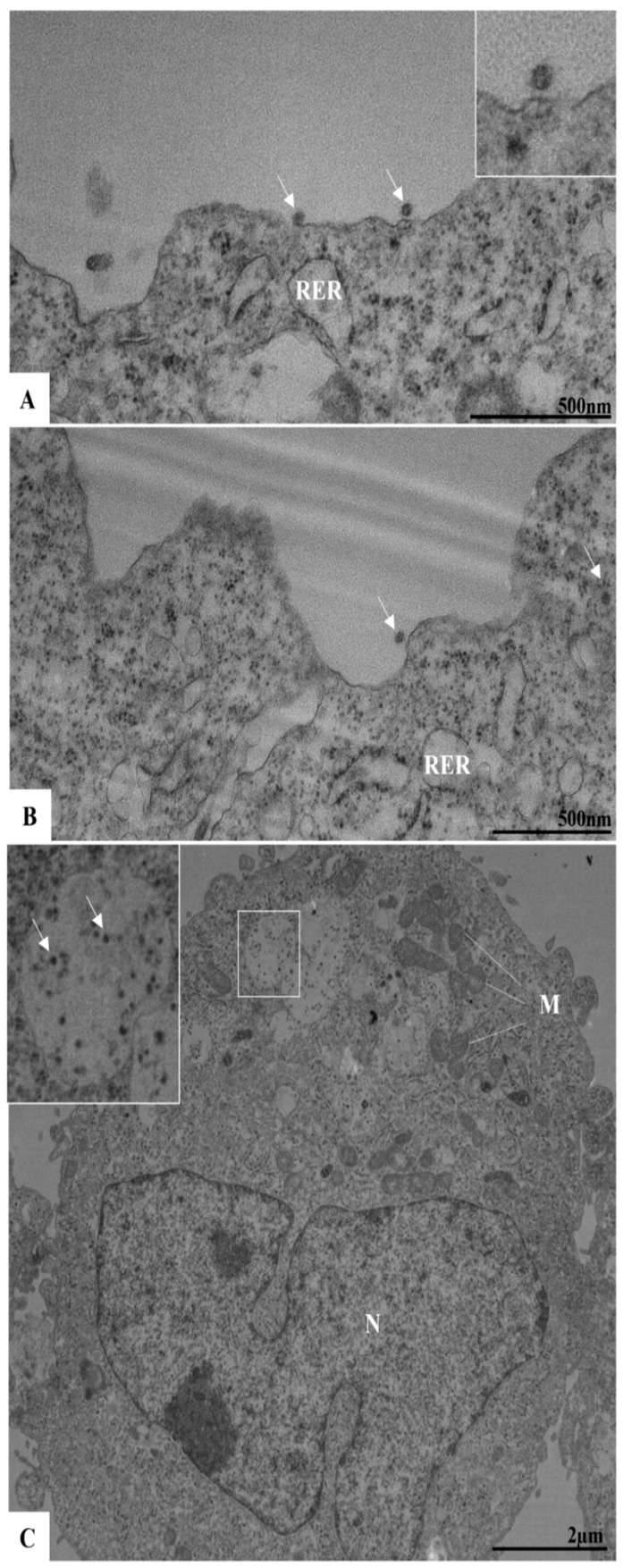
Viral particles in infected Vero cells in early times post-infection by ILHV (MOI 0.01). Virus particles (arrow) in adsorption in 1 h post-infection (**A**,**B**); several virus particles inside vesicles in cytoplasm in 3 h post-infection (**C**) [square]. Nucleus (N), mitochondria (M), rough endoplasmic reticulum (RER).

**Figure 5:**
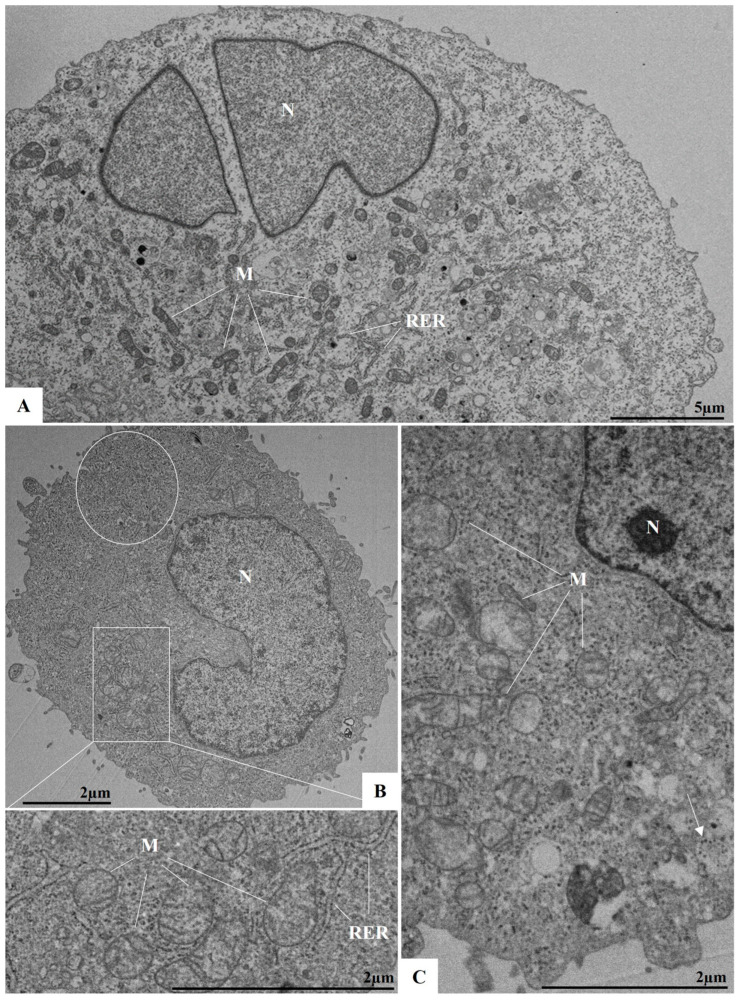
Ultrastructural alterations in infected Vero cells in early times post-infection by ILHV. (**A**) Uninfected Vero cells at 24 h of cultivation (cell control), (**B**) cells 2 h post-infection, and (**C**) cells 3 h post-infection. Note: electron-dense ribosomes (circle) (**B**), mitochondrial swelling and vacuolation (**B**,**C**), and virus particles inside vesicles (arrow). Nucleus (N), mitochondria (M), rough endoplasmic reticulum (RER).

To evaluate potential ultrastructural changes associated with ILHV infection during the early stages, and to observe events such as viral adsorption and replication, cells were analyzed at 1, 2, and 3 h p.i. At 1 h p.i., virus particles were observed near and attached to the plasma membrane, indicating the viral adsorption process (Figure 4A,B). At 3 h p.i., numerous viral particles were observed within vesicles (Figure 4C). From the second hour of infection onwards, more significant cellular alterations were evident, including the proliferation of the RER, the appearance of structures resembling viral factories, which are well-defined by membranes and surrounded by the RER, and the presence of electron-dense ribosomes and mitochondrial swelling and vacuolation (Figure 5B,C). Additionally, alterations such as double membrane vesicles (DMVs) and myelin figures were noted. No ultrastructural changes were observed in uninfected Vero cells (cell control, Figure 5A).

**Figure 6:**
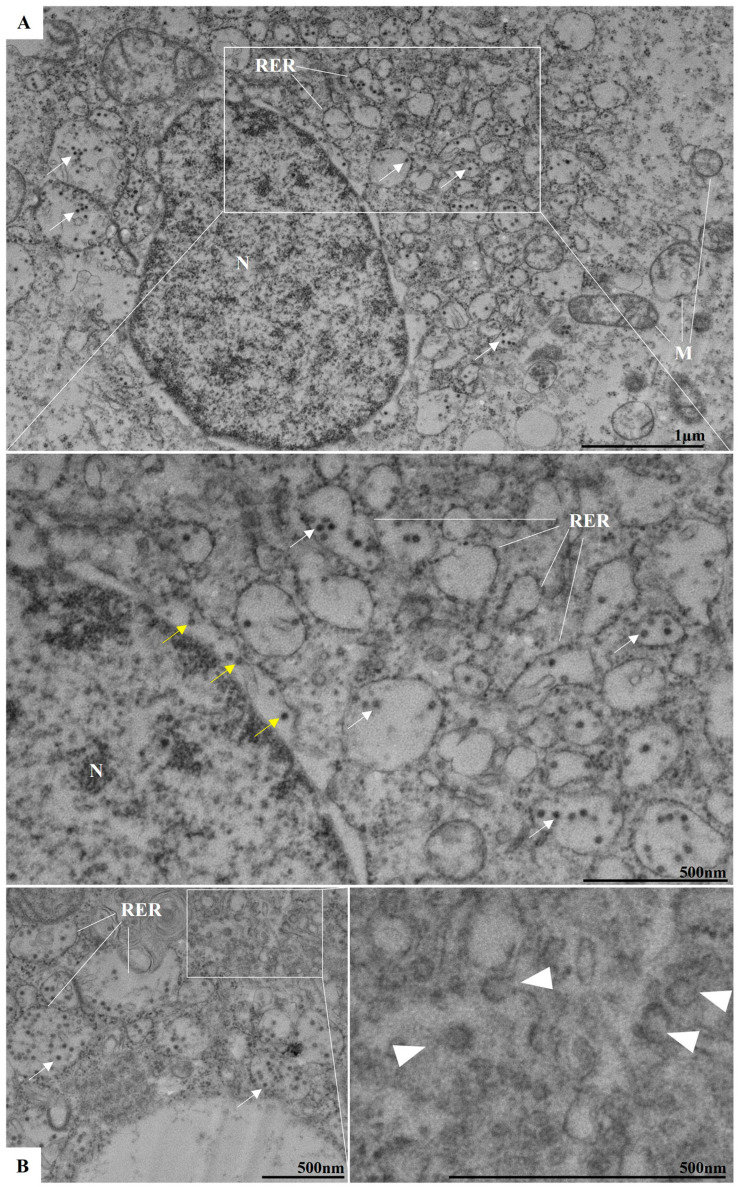
Proliferation of the rough endoplasmic reticulum (RER) in ILHV-infected Vero cells (48 h post-infection). Note: several virus particles inside RER cisternae (white arrow) (**A**,**B**), in perinuclear space (yellow arrow) (**A**), and sprouting areas near RER (arrowhead) (**B**). Nucleus (N), mitochondria (M).

In addition to the analyses conducted during the early stages of infection, studies at later time points (between 24 and 96 h p.i.) were also performed. At 48 h p.i., proliferation and thickening of the RER were observed. Consistent with other orthoflaviviruses, RER proliferation occurred concurrently with the presence of viral particles within this organelle (Figure 6A,B). Budding zones were identified near the cisternae of the RER (Figure 6B), where viral particles were localized. Viral particles were also detected in the perinuclear space (Figure 6A,B). Changes observed during the early stages of infection, such as the presence of electron-dense ribosomes and myelin figures, and loss of mitochondrial integrity, became more pronounced at later time points.

**Figure 7:**
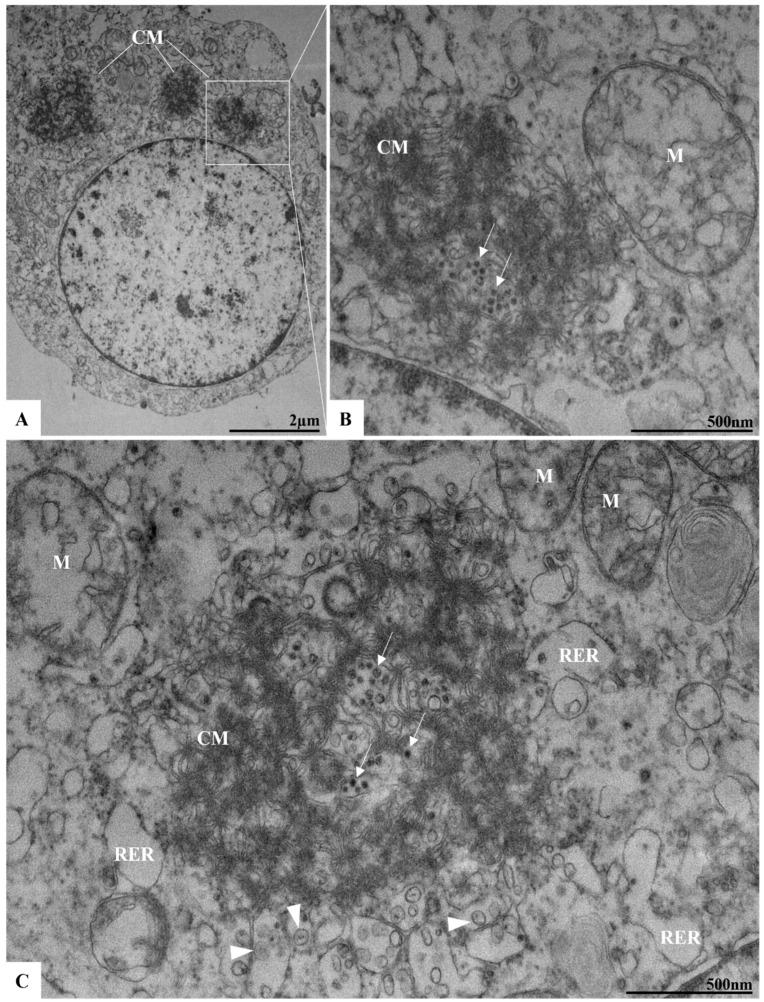
Convoluted membranes in Vero cells 72 h post-infection with ILHV. Presence of vesicle packages (arrowhead) near convoluted membrane (CM) (**A**,**B**). Incomplete virus particles (arrow) (**B**,**C**), mitochondria (M), nucleus (N), rough endoplasmic reticulum (RER).

**Figure 8:**
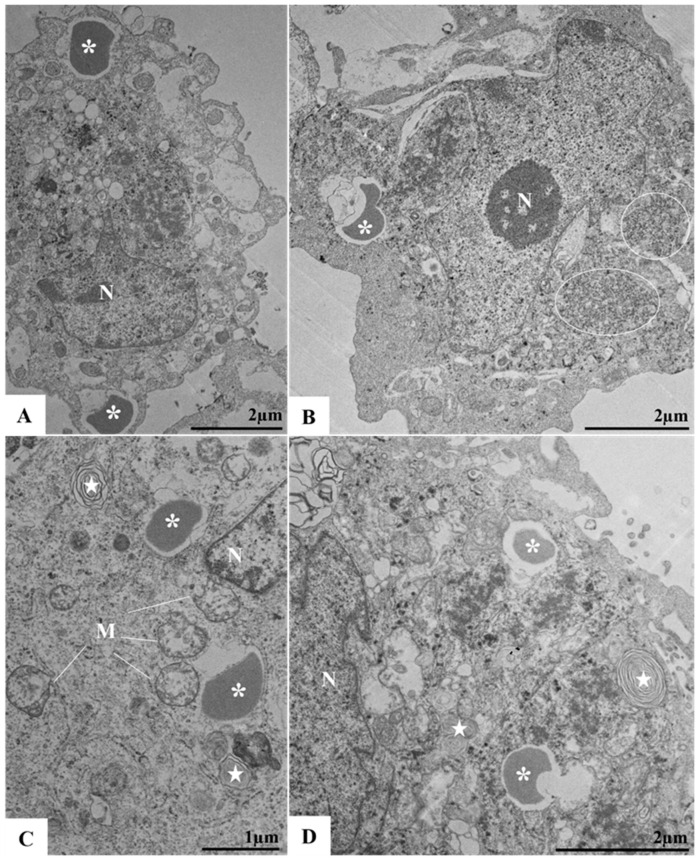
Proliferation of lipid droplets and presence of myelin figures in Vero cells infected by ILHV (96 h post-infection) (**A**–**D**). Areas with ribosomes thickening (circle) (**B**) and mitochondrial swelling and vacuolation (M) (**C**). Lipid droplets (*) (**A**–**D**), myelin figures (star) (**C**,**D**), nucleus (N).

Convoluted membranes (CMs) were observed in 72 and 96 h p.i. (Figure 7A–C). The CMs were encircled by cisternae of the RER, with incomplete viral particles localized within these structures. In association with the CMs and the RER, structures such as vesicular packets (VPs) were also identified (Figure 7C). The proliferation of lipid droplets was evident by 96 h p.i. (Figure 8A–C).

### 3.5. ILHV Particle Diameter

**Figure 9:**
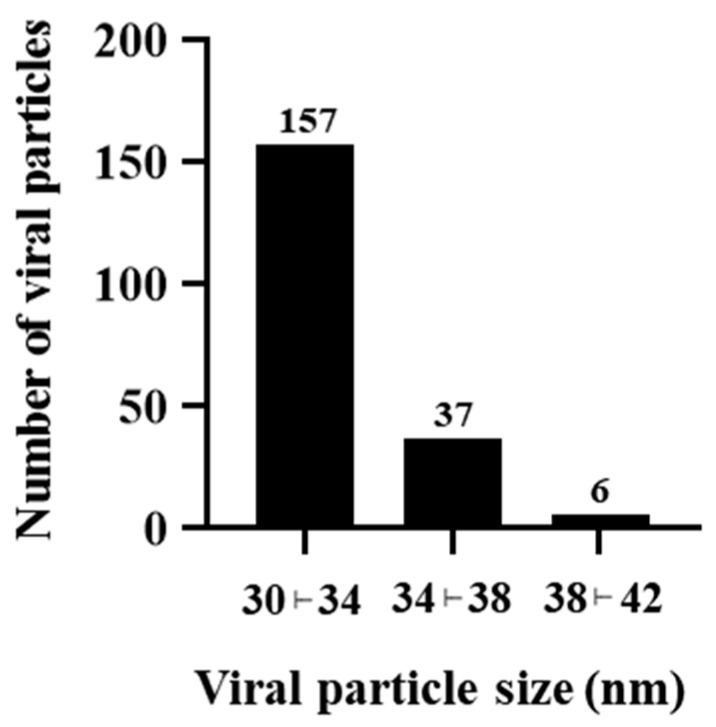
Histogram of viral particle size frequency distribution. Approximately 78% of the measured viral particles exhibited a diameter between 30 and 34 nanometers (nm).

Based on our data, the mean diameter of the viral particles was 32.45 nm with a standard deviation of 2.32 nm and a standard error of the mean (SEM) of 0.16 nm. The majority of virions were distributed within the frequency interval of 30 to 34 nm. However, the observed particle sizes ranged from 30.12 to 41.84 nm (Figure 9).

## 4. Discussion

ILHV is an arbovirus with considerable potential to cause epidemics and perhaps epizootics, particularly in tropical and subtropical regions due to its transmission by *Culex* sp. mosquitoes [27]. Despite its growing public health significance, research on this virus remains limited, particularly regarding the development of in vitro systems for antiviral drug screening and repurposing studies. The lack of robust experimental models hinders progress in understanding the viral infection mechanisms and developing effective therapeutic strategies. This underscores the urgent need for further research in this domain to address the growing threat posed by ILHV.

The susceptibility of different cell lineages to ILHV infection has been reported [27], but not all steps of the replication cycle have been fully elucidated.

Ultrastructural changes are commonly observed in cells in vitro studies that have been infected with different viruses. Particularly in infections by orthoflaviviruses, these alterations are related to organelles such as mitochondria, RER, and the Golgi complex. In infections by other viral entities like dengue and hepatitis C viruses, the formation of RER-derived membrane structures is one of the effects commonly observed [34]. Some of these formations include CM, DMVs, and VPs. In this study, we characterized these main morphological changes observed through both bright-field microscopy and TEM in Vero CCL81 cell line. The results highlight the susceptibility and permissiveness of the cell lineage to ILHV infection.

To observe the potential CPEs caused by ILHV infection, analyses were conducted using bright-field microscopy. Dengue and zika viruses are examples of other species classified to the same family that also induce CPE and cell death in different infected cell lines [35,36]. Our studies demonstrated that the Vero cell line infected by ILHV, exhibits classic CPE as observed with others arboviruses, especially from the second day of infection onwards, corroborating the data from McCormick et al. [36] and Offerdahl et al. [37].

Our ultrastructural evaluation by TEM showed viral particles near and attached to the plasma membrane, indicating that the adsorption process occurs within the first hour of infection. After 3 h of infection, numerous particles were observed in the lumen of the RER cisternae, which allows us to infer that viral replication was occurring.

Our morphometrical analysis demonstrated that the mean diameter of the viral particles was 32.45 nm, but diameter values reached up to 41 nm. These findings are consistent with other reports that described ILHV particles with an approximate diameter of 40 nm [19]. Moreover, other orthoflaviviruses have been described with diameters ranging from 40 to 50 nm [37,38].

Like other orthoflaviviruses, the replication of ILHV appears to be closely related to the RER. In our analysis, the proliferation of this cellular compartment occurred concurrently with the observation of viral particles in its lumen. In addition to proliferation, there was a thickening of this organelle. In regions adjacent to the RER, we also observed DMVs, VP, and CM. During the replication of orthoflaviviruses, structures known as replication complexes are formed, which are essential to support the assembly of the virions. Replication complexes are primarily derived from the RER; examples of compartments include VP and CM [21,39,40]. VPs have been recognized as the loci for viral genome replication [41]. CMs have been defined as structures for processing and storing viral polyproteins [22].

The conditions for the appearance of these structures in cells infected by different orthoflaviviruses are not yet well understood. Our results corroborate other studies demonstrating these CMs were described and associated with the RER in ILHV infection [19]. Moreover, viral particles were observed both inside the CMs and around these structures in the RER. Associated with the CMs, VPs were also described. VPs are formed by membrane invaginations that occur in the RER cisternae [39]. It is estimated that NSPs form replication structures that communicate with the cytoplasm through small pores. All these observed structures, including CMs, DMVs, and VPs, are related to virion synthesis and the replication process in cells.

In addition to the presence of CMs, DMVs, and membrane alterations resulting from viral infection, our findings also revealed the presence of lipid droplets at 96 h p.i. Lipid droplets are organelles with crucial roles in energy metabolism. During viral infections, these structures have been associated with key functions in the replication cycle and pathogenesis of viruses such as zika, hepatitis C, and the novel coronavirus [42,43,44]. In ILHV infection, lipid droplets interact with CMs, DMVs, and VPs, forming large replication complexes that contribute to pathogenesis and the assembly of virions.

In this study, our group demonstrated that Vero cells are susceptible and permissive to ILHV infection. Subsequently, we described the main morphological and ultrastructural changes resulting from viral infection. Thus, ILHV infection induces various types of membrane alterations, primarily those derived from the RER. CM, for example, may be involved in the replication cycle of these viruses, as well as DMVs. Myelin figures, on the other hand, are usually associated with membrane damage in organelles such as the mitochondria and the RER [45]. However, despite describing important characteristics of the ILHV replication cycle, other aspects such as viral adsorption, budding, and additional stages of the cycle remain poorly understood. These results highlight the importance of membrane-derived cellular structures for the ILHV replication cycle, and they establish the Vero cell line as a suitable model for future studies with ILHV, by allowing access to characteristic aspects expected for the replication cycle of these orthoflaviviruses.

## Data Availability

Data are contained within the article.
